# Microscopic Polyangiitis Initially Presumed to Be Endocarditis

**DOI:** 10.1155/2020/7020595

**Published:** 2020-03-14

**Authors:** Takahiro Kaneko, Shunsuke Hino, Yosuke Iijima, Norio Horie

**Affiliations:** Department of Oral and Maxillofacial Surgery, Saitama Medical Center, Saitama Medical University, Saitama, Japan

## Abstract

The antineutrophil cytoplasmic antibody- (ANCA-) associated vasculitides (AAVs), which include fever of unknown origin (FUO), are rare diseases characterized by necrotizing inflammation of small blood vessels and the presence of ANCAs. Microscopic polyangiitis (MPA) is a subtype of the AAVs. Although the prevalence of AAVs has generally increased over the last 20 years, there have been rare reports from the dental and oral surgery field. In this article, we present a case of MPA suspected to be infective endocarditis (IE) following tooth extraction.

## 1. Introduction

Fever of unknown origin (FUO) can be caused by a wide group of diseases including infections, noninfectious inflammatory diseases, malignancies, and miscellaneous diseases [1]. However, diagnosing certain causes of FUO has remained difficult. The antineutrophil cytoplasmic antibody- (ANCA-) associated vasculitides (AAVs) are rare diseases included in FUO characterized by necrotizing inflammation of small blood vessels and the presence of ANCAs [2]. AAVs can be classified into three phenotypically distinct processes: microscopic polyangiitis (MPA), granulomatosis with polyangiitis (GPA), and eosinophilic granulomatosis with polyangiitis (EGPA). Two main types of ANCA can be detected in patients with AAV: leukocyte proteinase 3 (PR3) and myeloperoxidase (MPO) [2]. Patients with GPA are predominantly PR3-ANCA-positive, whereas those with MPA or EGPA are predominantly MPO-ANCA-positive [2]. These antibodies are checked not only at diagnosis but also in follow-up as disease activity markers [3].

The prevalence of AAV is estimated to be 46–184/million people, and the numbers of patients with AAV have generally increased [4]. There is a relatively high prevalence of AAV among middle-aged and elderly persons [5], affecting both sexes equally (quite unlike most systemic autoimmune diseases) [5]. There is a geographic difference in the occurrence of AAVs.

Recently considered risk factors for AAVs include ultraviolet radiation and silica, which is commonly inhaled [4]. Although there have been studies that suggested infection as a risk factor, the long-held view that there is an infectious trigger has not been borne out [4].

The clinical presentation of AAVs begins with fever and weight loss, followed by systemic vasculitis characterized by renal and respiratory dysfunction [6]. In cases of MPA, necrotizing glomerulonephritis occurs rapidly, and it frequently progresses to renal failure [6]. Laboratory tests show increased C-reactive protein (CRP) and serum creatinine levels with increased MPO-ANCA [7]. In GPA, hemorrhages of the lung and upper respiratory tract and destructive lesions of the ear, nose, and throat are prominent [8]. EGPA patients typically have a long-standing history of asthma and allergic rhinitis [9].

Induction therapy for AAVs includes treatment with high-dose glucocorticoids (GCs) in combination with another immunosuppressive agent such as cyclophosphamide (CYC) or rituximab (RTX) [3]. RTX is more effective than CYC in refractory AAVs [3]. Patients with AAVs presenting with severe renal failure may be treated with pulsed CYC and GCs, with adjuvant plasma exchange [3].

Patients with FUO may visit dental or oral surgery clinics following recent dental treatment or have discomfort in the oral cavity. In this article, a rare case of MPA that was first referred to an oral surgery clinic for postextraction fever with dysgeusia and numbness of the tongue was presented. Initially, the authors suspected that the patient had infective endocarditis (IE) and thus consulted with specialists in cardiac medicine. However, IE was ruled out, with further detailed evaluations determining the presence of MPA.

## 2. Case Report

A 75-year-old woman was referred to the oral surgery clinic for a suspected dental infection. Her medical history included tuberculosis, hypertension, hyperlipidemia, and neurogenic bladder. Her surgical history included hip joint surgery at the ages of 11, 27, and 68 years. Four weeks earlier, the patient visited an otolaryngologist for otorrhea of the right ear and was diagnosed with otitis media and treated with antibiotics. On the same day, the patient underwent extraction of the right mandibular canine and lateral incisor by the patient's family dentist. Two weeks earlier, the patient had visited the primary care doctor for regular follow-up and complained of fatigue, fever, dysgeusia, swelling and numbness of the neck, and backache, which continued after the extraction. On laboratory examination, C-reactive protein (CRP) was 11.46 mg/dL, and the white blood cell count was 11,000/*μ*L. Subsequently, chest X-ray and computed tomography (CT) showed no abnormal findings, and she tested negative for influenza virus. The patient then developed trismus and pain at the apex of the tongue and was referred to the oral surgery clinic.

**Figure 1:**
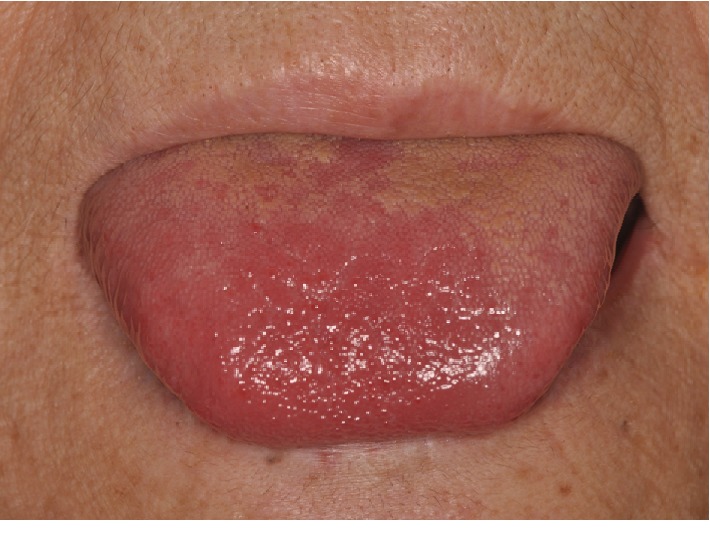
The apex of the tongue showing slight redness.

**Figure 2:**
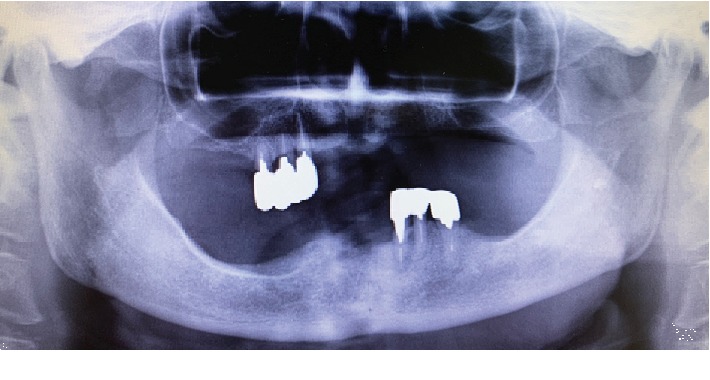
Orthopantomography at the first examination showing the sockets of the right mandibular canine and lateral incisor.

On examination, the patient complained of fever and discomfort of the tongue with dysgeusia. Although usually both the maxilla and mandible cause dentures to wear, this does not occur when the teeth have been extracted. Salivary function was relatively good, and there was no xerostomia observed. No trismus was found; the teeth extraction sockets had healed well. None of the other residual teeth exhibited any prominent caries or periodontal disease. There were no abnormal findings in the orofacial region except for the dysgeusia and redness and numbness at the apex of the tongue (Figure 1). Orthopantomography revealed no abnormal findings, including maxillary sinuses and temporomandibular joints (Figure 2). On physical examination, the patient's temperature was 38.5°C, and blood pressure was 125/75 mmHg. The serum CRP concentration was 19.4 mg/dL, and the WBC count was 14,700/*μ*L (Table 1). Given the history of tooth extraction, the patient was immediately referred to the division of cardiology for suspected IE.

The cardiologist took blood cultures, which were negative for bacteria, and an ultrasound cardiogram and transesophageal echocardiography showed neither clear vegetations nor regurgitation. For the remittent fever of more than 38.0°C, the patient was admitted to the cardiology clinic three days after the first visit and underwent a complete examination. Serum examination showed the following: PR3‐ANCA < 1 IU/mL, MPO‐ANCA 124.0 IU/mL, cytomegalovirus complement fixation test 8, ferritin 471 ng/mL, and CH50 60.0 U/mL. Sputum cultures, acid-fast bacterial cultures, and fecal cultures were negative. CT imaging showed no abnormal findings causing fever, and deep vein thrombosis was not found on ultrasonography of the veins of the lower extremities. There were no active symptoms related to the right otitis media other than depression of the eardrum on otolaryngological examination.

Fourteen days after the first visit, the patient was transferred to the Department of Rheumatology and Clinical Immunology because of the high suspicion of AAV, although renal dysfunction and findings of interstitial pneumonia were not evident. Twenty-seven days after the first visit, a renal biopsy was planned, but it was postponed due to influenza A infection. After the renal biopsy, which showed the findings of necrotizing glomerulonephritis, the patient was finally diagnosed with MPA (fever, MPO-ANCA-positive, CRP elevation, proteinuria, and histopathological findings of renal biopsy) 40 days after the first visit. Steroid therapy was then started with 30 mg prednisolone/day without immunosuppressive agents, and four days later, the patient was discharged. The inflammatory symptoms improved well, and the dysgeusia and numbness at the apex of the tongue disappeared four weeks later. At 20-week follow-up, prednisolone was tapered to 10 mg/day, and she was MPO-ANCA-negative.

The protocol of the report has been approved by the ethical review board of the university.

## 3. Discussion

To finally diagnose cases of FUO, detailed medical examination with various investigations and a longer time is needed. Dentists and oral surgeons should refer patients with fever to a physician immediately when the odontogenic diseases do not appear related to the cause of the fever. In addition, other differential diagnoses with FUO that may be initially encountered include Castleman's disease and adult-onset Still's disease [10].

In the present study, it seemed appropriate to initially diagnose the patient with suspected IE, because dentists are extremely well aware of the association between tooth extraction and IE. IE is defined as an infection of a native or prosthetic heart valve, the endocardial surface, or of an indwelling cardiac device. In this case, it was obviously important to refer the patient to a physician for meticulous examination. However, it is of note that there were oral symptoms that were overlooked and might suggest the occurrence of an AAV. The patient showed dysgeusia that had lasted for four weeks. AAVs frequently affect the peripheral and central nervous systems [11], and there was a case report of MPA that described multiple cranial neuropathies including dysgeusia [12]. As a matter of fact, diseases with both continuous fever and dysgeusia are few [13]. The patient felt numbness at the apex of the tongue. Numbness of the extremities and face has been reported in AAVs [14]. The numbness of the tongue may also be a sensory symptom caused by AAVs. In the present case, the dysgeusia and numbness of the tongue improved with the steroid therapy for MPA.

The patient had developed otitis media just before the tooth extractions. There are cases of AAVs that are complicated with otitis media, and the concept of otitis media with ANCA-associated vasculitis (OMAAV) has been proposed [15]. The criteria of OMAAV include otitis media with effusion or granulation resistant to antibiotics and insertion of tympanic ventilation tubes. It is unclear whether the otitis media in the present case could be considered OMAAV, because it improved before steroid therapy.

GPA, which was formerly known as Wegener's granulomatosis, has characteristic oral lesions of gingival swellings and nonspecific ulcerations, which are identified in 6-13% of cases [16]. However, there has been no report describing the other characteristics of AAV in the oral cavity. The dysgeusia and numbness of the tongue in the present case might be manifestations of AAVs that can be found in the oral cavity.

The patient's medical history revealed there were several previous issues, including tuberculosis, hypertension, hyperlipidemia, and neurogenic bladder. However, there was no indication of any association between these diseases and the AAVs.

## 4. Conclusion

The prevalence of AAVs has generally increased over the last 20 years, and AAVs are associated with high morbidity or mortality, especially if not immediately diagnosed and treated [4, 17]. Therefore, dentists and oral surgeons should keep AAVs in mind. At the same time, there may be hidden oral signs, such as the dysgeusia and numbness of the tongue in the present case, that may help make the diagnosis.

## Figures and Tables

**Table 1:** Laboratory data of the first examination.

		Normal range			Normal range
Complete blood cell count					
WBC (×10^3^/*μ*L)	14.7	3.25–8.59	Neutrophils (%)	91.7	40.30-71.70
RBC (×10^6^/*μ*L)	4.29	3.58-4.90	Hemoglobin (g/dL)	12.7	11.10–15.50
Platelets (×10^4^/*μ*L)	530	148.00–336.00			
Blood chemistry					
TP (g/dL)	6.5	6.50-8.00	ALB (g/dL)	2.6	3.70–5.00
CPK (U/L)	29	0.00-140.00	AST (U/L)	61	8.00–38.00
ALT (U/L)	53	4.00–43.00	LDH (U/L)	255	115-280
ALP	426	115.00-340.00	*γ* GTP (U/L)	44	10.00-47.00
Creatinine (mg/dL)	0.70	0.30–1.00	BUN (mg/dL)	8	5.00–22.00
UA mg/dL	1.4	2.00–6.20	eGFRcreat	61.3	90.00–999.90
Na (mEq/L)	138	136.00–145.00	Cl (mEq/L)	99	98-109
K (mEq/L)	3.6	3.30–5.00	Ca (mg/dL)	8.2	8.30–10.20
Zn (*μ*g/dL)	57	65.00-110.00	T-Bil (mg/dL)	0.5	0.20–1.00
CH50	60	31.60–57.60	CRP (mg/dL)	19.4	0.00–0.30
Procalcitonin (ng/mL)	0.1	0.00–0.40	cTnI (ng/mL)	0.00	0.00-0.09
Ferritin (ng/mL)^∗^	471	8.00–120.00	ASO^∗^ (IU/mL)	18	0.00–140.00
RF (IU/mL)^∗^	<11	0.00–15.00			
Immune tests					
*β*-D-Glucan (pg/mL)	≤5.0	0.00–10.90	ANA	80	0.00–80.00
Cryoglobulin	(-)	(-)	PR3-ANCA (U/mL)^∗^	<1	0.00–3.40
MPO-ANCA (U/mL)^∗^	124.0	0.00–3.40			
Viral tests					
CMV-CF	8	0.00–3.00	EB Anti-VCA/IgM-FA	<10	0.00-9.00
Tumor markers					
CEA-FEIA (ng/mL)^∗^	2.3	0.00–6.70	CA19-9 (U/mL)^∗^	10	0.00-37.00

These data include results of both examinations performed at the Oral and Maxillofacial Clinic and the Internal Medicine Clinic. ^∗^Data obtained later (day six to day ten after the first visit). WBC: white blood cell (count); RBC: red blood cell (count); TP: total protein; ALB: albumen; CPK: creatine phosphokinase; AST: aspartate aminotransferase; ALT: alanine aminotransferase; LDH: lactate dehydrogenase; ALP: alkaline phosphatase; *γ* GTP: *γ* glutamyl transpeptidase; BUN: blood urea nitrogen; UA: uric acid; eGFRcreat: estimated glomerular filtration rate based on serum creatinine; T-Bil: total bilirubin; CH50: 50% hemolytic complement activity; CRP: C-reactive protein; cTnI: cardiac troponin I; ASO: anti-streptolysin O antibody; RF: rheumatoid factor; ANA: antinuclear antibody; PR3-ANCA: proteinase 3-antineutrophil cytoplasmic antibody; MPO-ANCA: myeloperoxidase-antineutrophil cytoplasmic antibody; CMV-CF: cytomegalovirus complement fixation test; EB Anti-VCA/IgM-FA: Epstein-Barr antiviral capsid antigens/IgM fluorescence assay; CEA-FEIA: carcinoembryonic antigen fluorometric enzyme immunoassay; CA19-9: carbohydrate antigen 19-9.
